# Differential Patterns of Infection and Disease with *P. falciparum* and *P. vivax* in Young Papua New Guinean Children

**DOI:** 10.1371/journal.pone.0009047

**Published:** 2010-02-04

**Authors:** Enmoore Lin, Benson Kiniboro, Laurie Gray, Stuart Dobbie, Leanne Robinson, Annemarie Laumaea, Sonja Schöpflin, Danielle Stanisic, Inoni Betuela, Melinda Blood-Zikursh, Peter Siba, Ingrid Felger, Louis Schofield, Peter Zimmerman, Ivo Mueller

**Affiliations:** 1 PNG Institute of Medical Research, Madang, Papua New Guinea; 2 Centre for Global Health & Disease, Case Western Reserve University, Cleveland, Ohio, United States of America; 3 Walter & Eliza Hall Institute, Parkville, Victoria, Australia; 4 Department of Medical Biology, University of Melbourne, Melbourne, Victoria, Australia; 5 Swiss Tropical Institute, Basel, Switzerland; Singapore Immunology Network, Singapore

## Abstract

**Background:**

Where *P. vivax* and *P. falciparum* occur in the same population, the peak burden of *P. vivax* infection and illness is often concentrated in younger age groups. Experiences from malaria therapy patients indicate that immunity is acquired faster to *P. vivax* than to *P. falciparum* challenge. There is however little prospective data on the comparative risk of infection and disease from both species in young children living in co-endemic areas.

**Methodology/Principal Findings:**

A cohort of 264 Papua New Guinean children aged 1-3 years (at enrolment) were actively followed-up for *Plasmodium* infection and febrile illness for 16 months. Infection status was determined by light microscopy and PCR every 8 weeks and at each febrile episode. A generalised estimating equation (GEE) approach was used to analyse both prevalence of infection and incidence of clinical episodes. A more pronounced rise in prevalence of *P. falciparum* compared to *P. vivax* infection was evident with increasing age. Although the overall incidence of clinical episodes was comparable (*P. falciparum*: 2.56, *P. vivax* 2.46 episodes / child / yr), *P. falciparum* and *P. vivax* infectious episodes showed strong but opposing age trends: *P. falciparum* incidence increased until the age of 30 months with little change thereafter, but incidence of *P. vivax* decreased significantly with age throughout the entire age range. For *P. falciparum*, both prevalence and incidence of *P. falciparum* showed marked seasonality, whereas only *P. vivax* incidence but not prevalence decreased in the dry season.

**Conclusions/Significance:**

Under high, perennial exposure, children in PNG begin acquiring significant clinical immunity, characterized by an increasing ability to control parasite densities below the pyrogenic threshold to *P. vivax*, but not to *P. falciparum*, in the 2^nd^ and 3^rd^ year of life. The ability to relapse from long-lasting liver-stages restricts the seasonal variation in prevalence of *P. vivax* infections.

## Introduction

The epidemiology of *P. falciparum* malaria suggests immunity might be acquired in ‘stages’, with children first acquiring immunity against severe disease after relatively few episodes/infections [1], [2]. Uncomplicated *falciparum* disease, on the other hand, remains common throughout most of childhood and a significant decrease in risk of infection is only seen in adolescence and early adulthood [1], [3], [4], [5]. Similar age-dependent patterns of disease are also observed for *P. vivax*. In highly endemic areas, such as New Guinea, the risk of severe *P. vivax* disease is highest among children less than 2 yrs of age [6], [7], while uncomplicated *P. vivax* illness is rare among children over 5 yrs [5], [6] even though infections are commonly found even in adolescents and adults [3], [8], [9], [10]. Interestingly, compared to *P. falciparum*, the prevalence of infection with *P. vivax* peaks at younger ages [3], [8], [9], [10], contributes proportionally less to the burden of febrile illness [11] in older children, adolescents and adults and the risk of severe complications decreases faster with age [6], [7].

Differences in the epidemiology of *P. falciparum* and *P. vivax* have also been described in neighboring Vanuatu [12] and other areas of the world where the species co-exist [13], [14]. These epidemiological observations are consistent with data from malaria therapy patients that showed that immunity to *P. vivax* is more rapidly acquired than immunity to *P. falciparum* [15]. Whereas a single infection with *P. vivax* resulted in a strongly reduced incidence of febrile episodes upon homologous and to a lesser extent heterologous re-infection [16], most secondary *P. falciparum* infections were associated with fever, and in some cases high density parasitemia, even if re-infected with the same strain [17]. Similar patterns were observed under natural conditions in Sri Lankan patients [18].

Together, all these data indicate that under natural, lifelong exposure, immunity to *P. vivax* is acquired more quickly than immunity to *P. falciparum* and that different mechanisms may be responsible for protection against severe disease than those that protect against infections per se and mild episodes of disease [1], [19]. Although many potential targets and mechanisms of protective immunity have been identified for both *P. falciparum* [1], [19], [20], [21], [22] and *P. vivax* [23], [24], [25], we still know little about the actual mechanisms involved in the acquisition of protective immunity against either species and how these relate to the observed difference in the speed of immune acquisition.

A recent study in Papua New Guinean children 5–14 yrs of age found that despite acquiring a similar number of new blood stage infections with *P. falciparum* and *P. vivax*, children had attained very high levels of clinical immunity to *P. vivax* but remained at significant risk of uncomplicated *P. falciparum* illness [5]. This was linked to a greater ability to control *P. vivax* parasitaemia at levels well below the pyrogenic threshold than for *P. falciparum*. The control of parasite densities also increased with age in *P. falciparum*, but a significant reduction in risk was only evident for high density and symptomatic infections.

Immunological studies conducted as part of this cohort study showed that the control of *P. vivax* infections was linked to the acquisition of antibodies against the *P. vivax* Duffy binding protein (*Pv*DBP). In particular, children with high levels of blocking antibodies to region 2 of PvDBP had a 55% reduced risk of acquiring light-microscopy positive *P. vivax* infections [26]. Protection against clinical (and high density) *P. falciparum* infection was mediated by both humoral [27] (Richards, Mueller, Beeson, unpublished observations) and cellular immune responses [28], [29].

In order to describe the epidemiological patterns of infection and disease with *P. falciparum* and *P. vivax* and to identify factors associated with either risk or protection from infection and disease, we conducted a longitudinal cohort study of 264 Papua New Guinean children, aged 1–3 years at enrolment. The study design combined repeated blood sampling and molecular detection of parasitemia with a large array of classical and functional immune assays and thus provided an excellent opportunity to examine epidemiological evidence of different rates of acquisition of immunity to *P. falciparum* and *P. vivax* and investigate possible mechanisms of immune protection.

## Methods

This study was approved by institutional review boards of the PNG Medical Research Advisory Committee (Approval 05.19), University Hospitals Case Medical Center (Cleveland, Ohio USA), and the Swiss Tropical Institute.

### Field Site

**Figure 1:**
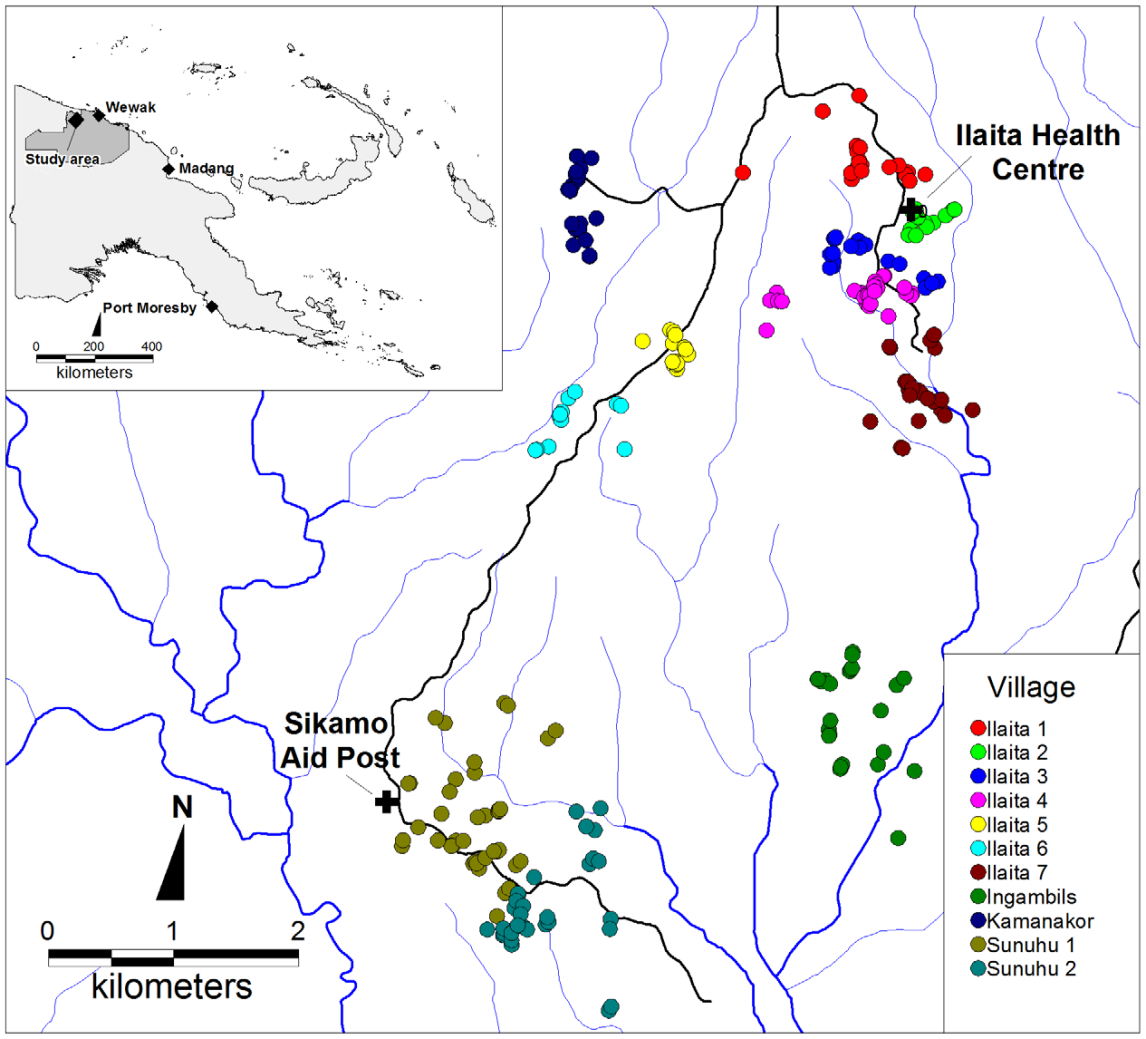
Schematic map of study area including participants' house (dots), health centres (crosses), roads (black lines) and rivers/streams (blue lines).

This study was conducted in 11 villages in the Ilaita area of Maprik District, East Sepik Province (Figure 1). Ilaita is situated at the northern edge of the Sepik River plain in the foot hills of the Torricelli Mountains, 80 km WSW of the provincial capital Wewak. The area is highly endemic for all 4 human malaria species, with *P. falciparum* the predominant parasite in all age groups except children ≤3 yrs where *P. vivax* is more common [10]. The study area is serviced by a single health sub-centre at Ilaita 2 run by the South Sea Evangelical Church Health Services and a government aid post at Sikamo near Sunuhu 1 (Figure 1). All villages are within less than 2 hrs walk (i.e. 5 km) of either health facility. Bednet use in the study area is heterogeneous, with coverage ranging from 0% (Sunuhu 2) to 98% (Ilaita 5).

### Enrolment

After obtaining community support and written parental consent, 190 children aged 1–3 yrs from Ilaita 1–7 and Sunuhu 1 and 2 were enrolled in March 2006. Demographic information was collected from all participating children and the location of each child's home was recorded using a hand-held GPS receiver (Garmin 12XL). Each child was clinically examined: axillary temperature was measured using digital thermometers, spleen palpated and a standard questionnaire of common signs and symptoms of malarial illness was administered. Hemoglobin (Hb) was measured using a portable device (HemoCue®, Ångholm, Sweden). A 5 ml venous blood sample was collected using Heparin-Vacutainer® tubes (Becton Dickinson, NJ, USA) and 2 blood slides (thick and thin films) made for determination of malarial infection. Bed net usage of both mother and child was queried. All children with parasitologically confirmed malaria (see below) were treated with Coartem® (arthemeter-lumefantrine); those with moderate to severe anaemia (i.e. Hb <7.5 g/dl) received an antimalarial treatment (Coartem®) plus 4 weeks of iron and folate supplementation according to national treatment guidelines.

As the initial enrolment did not yield the required sample size, the study area was extended to include Kamanokor and Ingamblis villages and 74 further children were enrolled into the study from the 1^st^ to the 3^rd^ regular bleed time points (May to September 2006).Enrolment procedures were identical with the exception of the 5 ml venous blood sample, which was not collected from these later enrolments. A 250 µl finger prick blood sample was collected instead.

### Active Follow-Up

After enrolment all children were actively followed-up for a maximum of 69 weeks. Every 2 weeks children were visited in their home villages and clinically examined using a standard questionnaire of signs and symptoms of malaria as well as a catalogue of danger signs based on the PNG approach to integrated management of childhood illness (IMCI). If the child was febrile (axillary temperature >37.5°C) and/or reported signs or symptoms of a febrile illness in the last 48 hrs or severe anaemia, a 250 µl finger prick blood sample was collected, two blood slides were made and Hb measured. An CIT Malaria (CIT Diagnostics, Cape Town, South Africa) rapid diagnostic test (RDT) was performed in all children with symptoms of malaria. All RDT results were confirmed by light microscopy within 24 hrs. All malaria RDT/light microscopy positive children were treated with Coartem®. All children with moderate-to-severe anaemia (Hb <7.5 g/dl) were given 4 weeks of iron and folate supplementation and in cases with positive blood films treated with Coartem®. Any children with signs of a severe illness were transported to the Ilaita Health centre or where appropriate to Maprik hospital. The guardians of all children were queried in regards to bednet use and illness episodes during the preceding 2 weeks. All reported illness episodes were cross-checked with the child's health book and dates and types of medication received by each child recorded.

In addition to fortnightly active surveillance for morbidity, all children were actively checked for the presence of malarial infection every 8 weeks using finger prick blood sampling. In order to improve the detection of low-level infections, children were visited on two consecutive days. If the child could not be found on the second day, the study team revisited the child's family 3 and/or 5 days after the first follow-up visit. On both days, a 250 µl finger prick blood sample was collected, two blood slides were made and Hb measured. A malaria RDT was only performed if the child was febrile and or reported signs or symptoms of a febrile illness. Children with parasitologically confirmed malarial illness or Hb <7.5 g/dl were treated in the same way as those detected during active morbidity surveillance. On the first follow-up day the child's spleen was palpated and the guardian queried in regards to bednet use and illness episodes during the preceding 2 weeks.

In total, children were followed for a maximum of 8 periods each spanning 3 fortnightly morbidity surveillance visits and an 8 weekly cross-sectional bleed visit. Due to public holidays (Easter, Independence week, Christmas, Easter) and the National General Election (July 2007), active follow-up had to be suspended for 5×1 week and 5 of 32 active follow-ups were thus conducted at a 3-week instead of a 2-week interval. As a consequence, 5 cross-sectional bleeds occurred at a 9 instead of 8-week interval. At the last cross-sectional bleed (i.e. week 69), the children were seen only once and a single 5 ml venous blood sample was collected instead of two consecutive finger prick samples.

### Passive Case Detection

During the entire follow-up period, a passive case detection system was maintained at the Ilaita health sub-centre and Sikamo aid post. All study children attending the outpatient clinic at Ilaita health centre were referred to specific study staff based at the health centre for clinical and parasitological assessment. Following a detailed clinical assessment, a 250 µl finger prick sample was collected, 2 blood slides made, Hb measured and a RDT performed in children showing signs or symptoms of malaria. Similar to ill children detected during active follow-up, only children with RDT or microscopy confirmed malaria illness were treated with Coartem®. Children with signs of severe illness were admitted to the health center inpatient wards or referred to Maprik Hospital. Study children attending Sikamo aidpost were attended to by the resident community health worker (CHW). Following a more basic clinical assessment, she performed RDTs on all suspected malarial cases and collected 2 blood slides and a filter paper blood spot. The CHW was instructed to only treat RDT positive children according to national treatment guidelines with Amodiaquine (3d) plus sulphadoxine pyrimethamine (stat) and refer negative children to either the IMR study team (if in the vicinity) or to the Ilaita health center for further investigation.

Despite good coverage of active and passive surveillance, a small number of children received treatment outside the study, either because they attended Ilaita health center after hours or because they sought treatment at a health facility outside the study area. All treatment dates and drugs given were entered into a specific database based on the children's health books and cross-checked with treatment information reported during active follow-up.

### Laboratory Methods

Plasma and peripheral blood mononuclear cells (PBMCs) were extracted from all venous blood samples. The remaining blood cells were pelleted and aliquoted accordingly. Finger-prick blood samples were separated into plasma and cell pellets. DNA was extracted using the QIAamp 96 DNA Blood kit (Qiagen Inc., Valencia, CA, USA) from the cell pellet fraction of all samples.

All research blood films were read independently by two expert microscopists. Slides with discrepant results were re-read by a 3^rd^ microscopist. Thick blood films were examined by light microscopy (LM) for 100 thick-film fields (under 100X oil immersion lens) before being declared infection-negative. Parasite species in positive films were identified and densities recorded as the number of parasites per 200 white blood cells (WBC). Densities were converted to the number of parasites per µl of blood assuming 8,000 WBC per µl (population average WBC count [3]). Slides were scored as LM-positive for an individual *Plasmodium* species, if the species was detected independently by at least 2 microscopists and/or subsequent PCR-based analysis confirmed the presence of the species. Densities were calculated as the geometric mean densities of all positive reads.

The presence of each of the four human malaria species was assessed in all blood samples using a semi-quantitative post-PCR, ligase detection reaction/fluorescent microsphere assay (LDR-FMA)[30]. This assay combines PCR amplification of the small subunit (ssu) ribosomal RNA gene (491–500 bp fragments) using genus specific primers, followed by a multiplex species-specific ligation detection reaction (LDR). The design and sensitivity of this assay has been described previously [9], [30], [31]. In order to guarantee maximum sensitivity for the detection of *Plasmodium* infections, the PCR-cycle number was set at 35. Cut-off values for positivity were set at the 99%-quantile of signals in negative controls as in Mueller et al [10].

Paired blood samples from 8-weekly cross-sectional double bleeds showed significant variance in the detection of malarial parasites both in LDR-FMA and LM diagnosis. In 35.3% of children infected with *P. falciparum* and in 27.4% of those infected with *P. vivax*, the infection was only detected by LDR-FMA in one of the two samples (data not shown). A comparable level of day-to-day variation in detection was observed in LM diagnosis. For those time points with blood samples on consecutive days, a combined diagnostic reading was calculated in the following way: i) a child was considered positive for any given *Plasmodium* species if it was positive on at least 1 day and ii) the density of the infection was defined as the higher of the two densities.

### Statistical Analyses

The analyses of both prevalence of infection and incidence of clinical episodes were based on a generalised estimating equation (GEE) approach that allowed accurate modelling of both the variation in the outcomes and the correlations between repeated measurements in individual children.

The prevalence of infection at the 8 or 9 weekly bleed time points were analysed using logistic GEE models via the XTLOGIT procedure in STATA 8.0. Preliminary analyses showed that the correlations between measurements in individual children were best modelled using an exchangeable correlation structure. A semi-robust Huber/White/sandwich estimator of variance was used to assure valid standard errors. Best fitting models were determined by backward elimination using Wald's Chi-square tests for individual variables. The analyses of age and spatial trend and predictors of infection were based exclusively on data from double bleed time points (i.e. weeks 9–60) using the combined diagnostic results from both samples (see above). As only one sample was collected at baseline and final bleeds (i.e. weeks 0 and 69), only prevalence data from the first sample collected at double bleed time points was used for the analyses of time trends across the entire time of follow-up. Difference in log-transformed parasite densities among age groups were analysed using a normal GEE model with exchangeable correlation structure and semi-robust variance estimator.

For estimation of incidence of clinical episodes in each 8 or 9 week follow-up interval, clinical malaria episodes were defined as a fever (i.e. axillary temperature >37.5°C) or history of fever during the last 48 hrs in the presence of a light microscopically detectable parasitaemia observed in either passive or active follow-up. Febrile episodes with only LDR-FMA detectable parasitaemia were not considered to be malarial episodes. A density cut-off value for clinical disease was set based on a pyrogenic threshold of 2500 parasites/µl for *P. falciparum* and 500 parasites/µl for all other species [32]. Moderate and high density episodes were defined as febrile episodes with parasitaemias exceeding the pyrogenic threshold 4 and 20-fold, respectively. For each interval, children were considered at risk from the 1^st^ day after the 2^nd^ or only blood sample was taken. Cross-sectional bleed days were thus considered part of the preceding interval and clinical episodes detected during the cross-sectional bleeds were included in that interval. Children were not considered at risk for 2 weeks after treatment with Coartem® and 4 weeks after treatment with AQ plus SP.

As preliminary analyses showed significant overdispersion in the number of episodes per child, a negative binomial model GEE model (based on XTNBREG procedure) was used for the analyses of incidence rates. As in the other GEE models, an exchangeable correlation structure and semi-robust variance estimator were used and best fitting models determined by backward elimination using Wald's Chi-square tests for individual variables. Differences in participant characteristics at enrolment were assessed using Chi-square and Fisher's exact tests. All analyses were done using the STATA 8 statistical software package (College Station, TX).

## Results

### Enrolment

**Figure 2:**
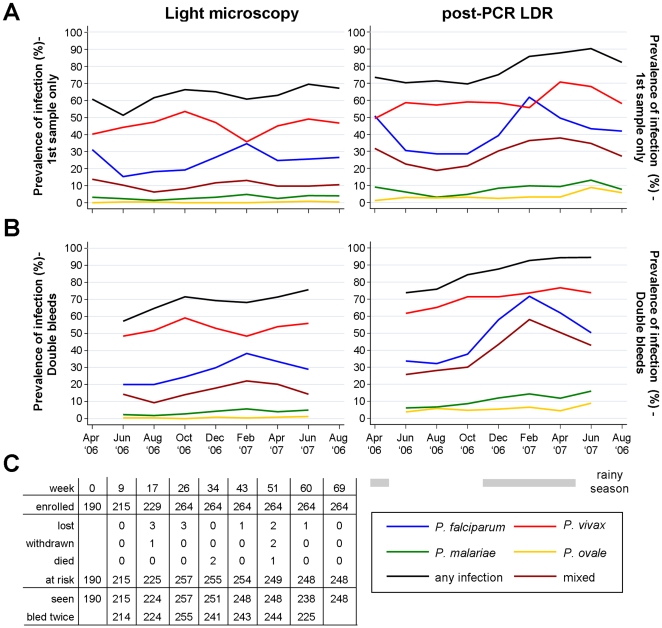
Prevalence of malaria at regular cross-sectional surveys: A) first samples only, B) double bleed samples 24 hrs apart, C) study retention profile.

Between March and September 2006, a total of 293 children 1–3 yrs of age (±3 months) were screened for inclusion into the study. Of these, 21 were excluded from enrolment due to chronic illness, permanent disability or because they were severely malnourished. An additional 8 children were excluded post enrolment either because their guardians withdrew consent within less than 8 weeks from enrolment (n = 6) and/or because they had very poor and erratic attendance (n = 2). Of the 264 children in the final cohort, 190 were enrolled at study start in March 2006 and 74 over the following 6 months (Figure 2).

At enrolment, children were between 0.91–3.21 yrs of age (median 1.70, Inter-quartile range (IQR): [1.27, 2.43] and 118 (43.9%) were female. There were no differences in demographic characteristics between early and later enrolled children (p>0.5). *Plasmodium* infections were diagnosed in 199 (75.4%) and 168 (63.6%) children by post-PCR LDR-FMA and light microscopy (LM), respectively. *P. vivax* was the most common parasite (LDR-FMA: 53.0% LM: 44.3%) followed by *P. falciparum* (LDR-FMA: 49.6% LM: 32.6%). *P. malariae* (LDR-FMA: 9.9% LM: 4.2%) and *P. ovale* (LDR-FMA: 2.7%, LM: 0.0%) were present but relatively rare. Mixed species infections were detected in 85/199 (42.7%) LDR-FMA and 45/168 (26.8%) LM positive children. There were no significant differences in the prevalence of *P. falciparum* infection between early and late enrolments (p>0.09). While later enrolments did have a significantly higher prevalence of *P. vivax* (LDR: 50.0% vs 63.5%, p = 0.033; LM: 40.0% vs 55.4%, p = 0.024) at the time of enrolment, the prevalence of *P. vivax* infection in late enrolled children was comparable to that observed in concurrently collected samples from earlier enrolled children (p>0.05).

A high degree of morbidity was observed during enrolment. Sixty-four children (23.9%) presented with an axillary temperature >37.5°C or were reported to be suffering from a febrile illness. Of these, 54 (85.94%) had concurrent LM-positive malarial infections of any density compared to 56.5% of afebrile children (Odds-ratio (OR): 4.71, CI_95_ [2.14, 11.38], p<0.001), with 44 (68.8%) exceeding 2500 parasites/µl for *P. falciparum* and 500/µl for non-*falciparum* infections (14 Pf, 21 Pv, 2 Pm and 7 Pf plus Pv). Mean haemoglobin level was 8.90 g/dl with 19.7% (52/264) of children diagnosed with moderate-to-severe anaemia (Hb <7.5 g/dl). Late enrolments were significantly more likely to suffer from fever (OR: 2.17 CI_95_ [1.14, 4.08.38], p = 0.01), malaria (any density OR: 2.23 CI_95_ [1.13, 4.32], p = 0.011) and/or anaemia (Hb <7.5 g/dl, OR: 2.78 CI_95_ [1.40, 5.46], p = 0.001) than early enrolled children. However, after adjusting for village of residence the observed difference in morbidity among early and later enrolled children was no longer statistically significant (p>0.1). Early and later enrolled children were thus combined for all further analyses.

Children were then followed up for up to 69 weeks. 248 of 264 (93.9%) children were retained until the end of the study, 10 children were lost during follow up and 3 were withdrawn by their parents (Figure 2). 3 children died during the conduct of the study, 1 from heart failure caused by severe malarial anaemia, 1 from severe malaria and 1 from dysentery, based on clinical diagnosis. Compliance with the study protocol was very high. 96.0%–100.0% of children at risk were seen at regular 8-weekly bleeds with 90.7%–99.6% of children seen on two consecutive days at the week 9 to 60 bleeds. Attendance at the 2-weekly active morbidity follow-up ranged from 88.6%–99.5%.

### Parasitaemia during Follow-Up

After enrolment a gradual increase in the prevalence of malarial infections was observed by both LDR-FMA and LM. There were, however, pronounced differences in both overall and seasonal trends between different *Plasmodium* species (Figure 2). While increasing significantly during the course of the study (non-parametric test for trend, p<0.01), *P. falciparum* prevalence was highly seasonal (1^st^ sample only, adjusted for significant linear trend: LDR-MFA: Χ^2^ = 89.8, df  = 5, p<0.001, LM: Χ^2^ = 28.6, df  = 5, p<0.001). By both methods of detection, *P. falciparum* prevalence decreased significantly during the dry season (May-Sept ‘06) and rose again rapidly with the onset of the rains in Nov 06 with *P. falciparum* peaking in Jan 07 (LDR: 61.8%, LM: 34.7%). Seasonal trends for *P. vivax* were much less pronounced. When diagnosed by LDR-FMA, *P. vivax* prevalence did show a significant increase over time (p<0.01) with only weak evidence for seasonality (Χ^2^ = 10.8, df  = 5, p = 0.057). By LM no trend for increasing prevalence was observed (p = 0.54), but *P. vivax* showed considerable seasonality (Χ^2^ = 20.8, df  = 5, p<0.001) with a significant increase in prevalence through the dry season peaking in Sept 06’ (53.5%) and a pronounced drop in prevalence in the early rainy season (Jan ’07: 35.8%). Among children with two consecutive samples, seasonal trends were even stronger for *P. falciparum* but less evident for *P. vivax* (Figure 2). By LDR-FMA, significant increases over time were also observed for *P. malariae* (p = 0.02) and *P. ovale* (p<0.01), with seasonal trends of *P. malariae* (Χ^2^ = 16.5, df  = 5, p = 0.005) and *P. ovale* (Χ^2^ = 10.8, df  = 5, p = 0.057) parallel to those exhibited by *P. falciparum* and *P. vivax*, respectively.

Besides time trends, there was also significant spatial variation in the prevalence of malarial infections (Tables 1 & 2). Prevalence rates were highest in villages furthest from the Ilaita Health centre (i.e. Sunuhu 1&2, Kamanokor, Ilaita 5 and Ingamblis). Spatial variation was more pronounced for *P. falciparum* (LDR-FMA: Χ^2^ = 58.9, df  = 10, p<0.001: LM: Χ^2^ = 36.3, df  = 10, p<0.001) compared to *P. malariae* (LDR-FMA: Χ^2^ = 23.6, df  = 10, p = 0.008; LM: Χ^2^ = 14.2, df  = 10, p = 0.14) and *P. vivax* (LDR-FMA: Χ^2^ = 19.9, df  = 10, p = 0.031: LM: Χ^2^ = 14.4, df  = 10, p = 0.16).

**Table 1:** Multivariate predictors of malarial infection at double bleed time points as diagnosed by post-PCR LDR-FMA assay.

	Any infection		*P. falciparum*		*P. vivax*		*P. malariae*		*P. ovale*	
	OR	CI_95_	OR	CI_95_	OR	CI_95_	OR	CI_95_	OR	CI_95_
**Village** 1										
Ilaita 2	0.55	[0.19,1.61]	0.47	[0.24,0.90]	0.50	[0.22,1.16]	3.94	[1.67,9.28]		
Ilaita 3	0.81	[0.28,2.37]	0.94	[0.52,1.68]	0.81	[0.38,1.75]	2.11	[0.78,5.74]		
Ilaita 4	0.74	[0.26,2.12]	0.91	[0.55,1.50]	0.55	[0.26,1.17]	2.04	[0.82,5.05]		
Ilaita 5	2.76	[0.86,8.87]	1.14	[0.55,2.38]	1.34	[0.55,3.30]	1.31	[0.46,3.77]		
Ilaita 6	0.64	[0.22,1.85]	0.50	[0.22,1.14]	0.82	[0.36,1.87]	1.59	[0.43, .91]		
Ilaita 7	0.85	[0.31,2.32]	1.00	[0.59,1.71]	0.53	[0.27,1.06]	2.03	[0.82,5.03]		
Ingamblis	3.18	[1.11,9.15]	1.77	[0.93,3.37]	1.11	[0.55,2.27]	4.43	[1.70,11.6]		
Kamanokor	4.65	[0.84,25.8]	3.36	[1.91,5.93]	0.93	[0.44,1.93]	4.42	[1.72,10.3]		
Sunuhu 1	2.04	[0.70,5.93]	2.04	[1.25,3.35]	0.84	[0.42,1.69]	3.04	[1.28,7.23]		
Sunuhu 2	1.88	[0.62,5.70]	1.89	[1.04,3.42]	1.14	[0.56,2.36]	2.30	[0.90,5.89]		
		p = 0.001		p<0.001		p = 0.031		p = 0.008		
**Month** ^2^										
March			0.62	[0.40,0.96]						
May			0.30	[0.21,0.43]						
July			0.22	[0.14,0.34]						
September			0.21	[0.14,0.33]						
November			0.44	[0.29,0.66]						
				p<0.001						
**Year (’07)**	2.83	[1.79,4.47]					1.44	[1.01,2.07]	0.70	[0.50,0.97]
		p<0.001						p = 0.046		p = 0.035
**Age**										
linear	62,791		10.1	[3.52,29.1]	4,898		1.43	[1.10,1.86]	1.35	[1.02,1.80]
quadratic	0.22		0.70	[0.58,0.86]	0.04			p = 0.008		p = 0.038
cubic	1.56			p<0.001	1.51					
		p<0.001				p<0.001				
**Prior Drug**	0.59	[0.41,0.85]	1.68	[1.27,2.22]	0.64	[0.48,0.84]				
		p = 0.005		p<0.001		p = 0.002				
**Ill 2 weeks**	0.57	[0.37,0.87]					0.55	[0.33,0.91]		
		p = 0.009						p = 0.021		
**ITN use** ^3^	0.43	[0.24,0.75]			0.62	[0.42,0.92]			0.51	[0.31,0.87]
		p = 0.003				p = 0.018				p = 0.013
**Anaemia**										
Hb (g/dl)^3^	0.59	[0.47,0.73]	0.66	[0.59,0.74]						
Hb ≥8 g/dl		p<0.001		p<0.001	2.44	[1.69,3.57]				
						p<0.001				
**Spleen**			1.47	[1.25,1.74]			1.47	[1.24,1.75]		
				p<0.001				p<0.001		
**Febrile**			1.63	[1.27,2.15]	0.62	[0.47,0.82]				
				p = 0.001		p = 0.001				

GEE-model based estimates with semi-robust confidence intervals.

OR: Odds ratio. CI_95_: 95% confidence intervals

1 Comparison level: Iliata 1. ^2^ Comparison level: January. ^3^ Average bed net usage as continuous variable. ^4^Per g/dl increase

**Table 2:** Multivariate predictors of malarial infection at double bleed time points diagnosed by light microscopy.

	Any infection		*P. falciparum*		*P. vivax*		P. malariae	
	OR	CI_95_	OR	CI_95_	OR	CI_95_	OR	CI_95_
**Village** 1								
Ilaita 2	0.58	[0.23,1.51]	0.50	[0.22,1.16]				
Ilaita 3	1.12	[0.47,2.68]	1.21	[0.67,2.18]				
Ilaita 4	0.66	[0.31,1.43]	0.78	[0.40,1.52]				
Ilaita 5	2.34	[0.80,6.79]	1.76	[0.70,4.38]				
Ilaita 6	0.84	[0.36,1.96]	0.61	[0.27,1.38]				
Ilaita 7	0.67	[0.32,1.39]	0.55	[0.24,1.23]				
Ingamblis	1.67	[0.76,3.65]	1.36	[0.70,2.65]				
Kamanokor	1.84	[0.77,4.36]	2.51	[1.43,4.40]				
Sunuhu 1	1.65	[0.80,3.43]	1.92	[1.16,3.18]				
Sunuhu 2	2.31	[1.10,4.86]	1.76	[0.98,3.16]				
		p<0.001		p<0.001				
**Month** ^2^								
March			0.77	[0.49,1.21]				
May			0.58	[0.39,0.86]				
July			0.62	[0.39,0.99]				
September			0.52	[0.33,0.81]				
November			0.83	[0.52,1.33]				
				p = 0.023				
**Age**								
linear	19.9	[6.11,65.1]	35.4	[10.1,124]	1.19	[1.00,1.42]		
quadratic	0.66	[0.52,0.83]	0.58	[0.46,0.74]		p = 0.049	1.22	[1.13,1.31]
		p<0.001		p<0.001				p<0.001
**Prior Drug**	0.22	[0.16,0.30]			0.30	[0.22,0.39]		
		p<0.001				p<0.001		
**Ill 2 weeks**	0.51	[0.36,0.73]			0.50	[0.37,0.67]	0.26	[0.09,0.76]
		p<0.001				p<0.001		p = 0.014
**ITN use**	0.52	[0.31,0.83]	0.62	[0.40,0.96]	0.53	[0.39,0.73]		
		p = 0.007		p = 0.033		p<0.001		
**Anaemia**								
Hb (g/dl) ^3^	0.58	[0.50,0.66]	0.52	[0.45,0.61]			0.64	[0.53,0.76]
Hb <8 g/dl		p<0.001		p<0.001	2.22	[1.52,3.23]		p<0.001
						p<0.001		
**Spleen**			1.22	[1.04,1.46]			1.80	[1.47,2.19]
				p = 0.018				p<0.001
**Febrile**	1.46	[1.00,2.08]	2.19	[1.60,3.00]				
		p = 0.047		p<0.001				

GEE-model based estimates with semi-robust confidence intervals.

OR: Odds ratio. CI_95_: 95% confidence intervals

1 Comparison level: Iliata 1. ^2^ Comparison level: January. ^3^ Average bed net usage as continuous variable. ^4^Per g/dl increase.

### Incidence of Clinical Disease

Over the 69 weeks of follow-up, a total of 1134 febrile episodes (incidence rate (IR) 4.60 / child / yr) with parasitaemia (by light microscopy) were observed. Of these 842 (IR 3.42) had a concurrent parasitaemia >2,500 for *P. falciparum* and >500 for *non-falciparum* infections. *P. falciparum* was the most common cause of malarial illness (any density: 630 (IR 2.56), Pf >2,500: 472 (IR 1.92), Pf >10,000: 379 (IR 1.54), Pf >50,000: 206 (IR 0.84)) followed by *P. vivax* (any density: 605 (IR 2.46), Pv >500: 391 (IR 1.59), Pv >2,000: 271 (IR 1.10), Pv >10,000: 113 (IR 0.84)). *P. malariae* (any density: 49 (IR 0.20)) and *P. ovale* (any density: 7 (IR 0.03)) episodes were rare. 161 of 1134 (14.2%) clinical episodes were the result of mixed species infections of any density. Of the 842 infections that exceeded species-specific pyrogenic density cut-offs, 59 were mixed infections (7.0%) where both species exceeded the density threshold. *P. falciparum* / *P. vivax* mixed infections accounted for 83.8% (135/161) of mixed infections of any density and 84.7% (50/59) of mixed infections that exceeded the pyrogenic parasitaemia thresholds. Detailed analyses on mixed species interactions and their effect on risk of morbidity will be presented elsewhere.

**Figure 3:**
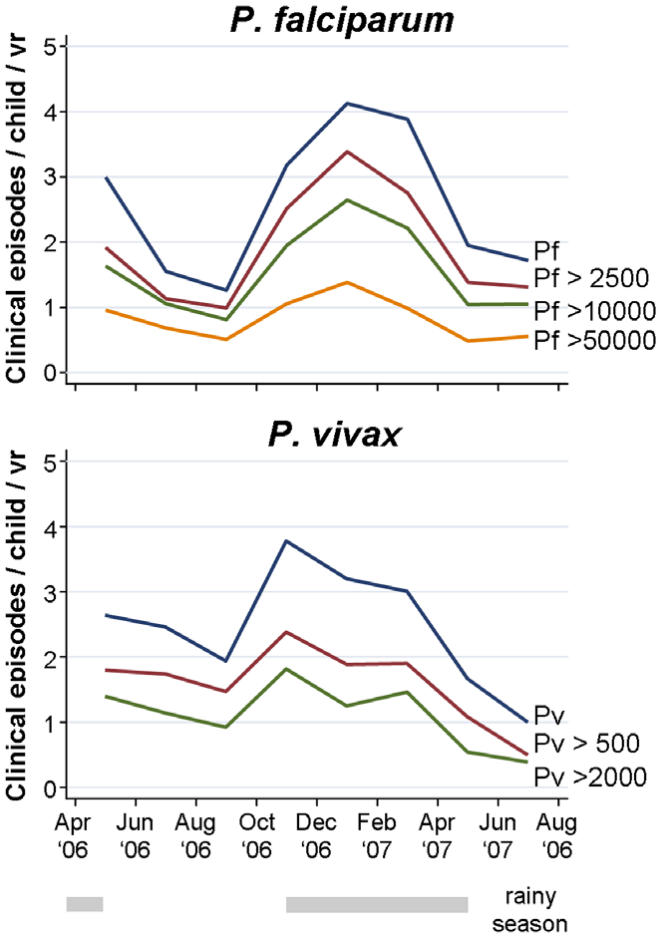
Incidence of *P. falciparum* and *P. vivax* clinical episodes with different parasitaemia threshold for individual 8/9 weekly intervals.

As seen for prevalence of infection, the incidence of malarial episodes showed strong seasonality (Figure 3, Table 3 & 4). In parallel with the prevalence of infections, incidence rates of *P. falciparum* episodes decreased significantly (any density: Χ^2^ = 101.9, df  = 5, p<0.001, Pf >2500: Χ^2^ = 90.5, df  = 5, p<0.001) during the dry season reaching its lowest level in the Aug-Sept ’06 period (any density: IR 1.27, Pf >2500: IR 0.99) before rising quickly following the onset of rains in October and peaking in Dec‘06–Jan ’07 (any density: IR 4.13, Pf >2500: IR 3.39) before falling once again towards the end of the rainy season in Apr - May ’07 (any density: IR 1.95, Pf >2500: IR 1.39). The seasonal trends for incidence of *P. vivax* episodes were comparable to those seen in *P. falciparum* although the incidence of *P. vivax* peaked earlier in the rainy season (Oct–Nov ’08, any density: IR 3.23, Pv >500: IR 2.38) before decreasing consistently during the remainder of the rainy and into the dry season (any density: Χ^2^ = 53.3, df  = 5, p<0.001, Pv >500: Χ^2^ = 30.0, df  = 5, p<0.001). Similar trends were observed in the incidence of *P. falciparum* and *P. vivax* episodes with higher parasite densities (Figure 3). The seasonal trends in *P. vivax* episodes were thus at least partially uncoupled from those seen for prevalence of *P. vivax* infection. No seasonal differences were observed for episodes of *P. malariae* (Χ^2^ = 2.2, df  = 5, p = 0.82).

**Table 3:** Multivariate predictors of incidence of infections in 8 × 8/9 weeks intervals: All and *P. falciparum* episodes of different parasite density.

	All episodes		Pf all		Pf >2,500		Pf >10,000	
	IRR	CI_95_	IRR	CI_95_	IRR	CI_95_	IRR	CI_95_
**Village** 1								
Ilaita 2	0.77	[0.51,1.16]	0.48	[0.25,0.93]	0.47	[0.22,1.01]	0.49	[0.23,1.06]
Ilaita 3	1.14	[0.80,1.66]	0.62	[0.37,1.04]	0.55	[0.31,0.97]	0.48	[0.27,0.84]
Ilaita 4	0.92	[0.68,1.25]	0.58	[0.37,0.89]	0.43	[0.26,0.72]	0.41	[0.24,0.70]
Ilaita 5	0.73	[0.52,1.03]	0.96	[0.63,1.46]	0.76	[0.48,1.20]	0.55	[0.34,0.91]
Ilaita 6	1.13	[0.76,1.68]	0.59	[0.32,1.09]	0.41	[0.19,0.88]	0.35	[0.16,0.78]
Ilaita 7	0.89	[0.67,1.18]	0.65	[0.43,0.99]	0.41	[0.24,0.67]	0.35	[0.20,0.63]
Ingamblis	0.55	[0.40,0.75]	0.42	[0.26,0.67]	0.34	[0.20,0.57]	0.25	[0.14,0.42]
Kamanokor	0.91	[0.67,1.23]	0.98	[0.66,1.46]	0.81	[0.50,1.31]	0.68	[0.42,1.11]
Sunuhu 1	0.83	[0.62,1.11]	0.78	[0.52,1.17]	0.68	[0.42,1.08]	0.51	[0.31,0.83]
Sunuhu 2	0.77	[0.57,1.05]	0.76	[0.50,1.15]	0.67	[0.41,1.07]	0.48	[0.29,0.78]
		p = 0.003		p<0.001		p<0.001		p<0.001
**Month** ^2^								
March	0.51	[0.41,0.63]	0.50	[0.39,0.65]	0.51	[0.38,0.70]	0.51	[0.36,0.72]
May	0.41	[0.34,0.50]	0.35	[0.26,0.46]	0.40	[0.28,0.56]	0.44	[0.30,0.65]
July	0.30	[0.23,0.41]	0.20	[0.13,0.30]	0.25	[0.15,0.41]	0.26	[0.15,0.43]
September	0.61	[0.46,0.81]	0.53	[0.37,0.75]	0.70	[0.46,1.08]	0.70	[0.43,1.14]
November	0.64	[0.48,0.85]	0.67	[0.48,0.93]	0.88	[0.58,1.32]	0.87	[0.56,1.35]
		p<0.001		p<0.001		p<0.001		p<0.001
**Year (’07)**	0.61	[0.50,0.74]	0.59	[0.45,0.78]	0.69	[0.49,0.98]	0.70	[0.50,0.97]
		p<0.001		p<0.001		p = 0.040		p = 0.035
**Age**								
linear			1.35	[1.18,1.54]	1.20	[1.03,1.41]		
quadratic				p<0.001		p = 0.021		
**Infection status** (at start of interval)								
Any	1.73	[1.36,2.20]						
Pf		p<0.001	1.23	[1.02,1.48]	1.35	[1.10,1.64]	1.43	[1.14,1.80]
				p = 0.028		p = 0.004		p = 0.002
**Prior drug**			0.78	[0.67,0.92]	0.77	[0.62,0.95]		
				p = 0.002		p = 0.015		
**ITN use** ^3^	0.77	[0.61,0.98]	0.59	[0.41,0.85]	0.58	[0.38,0.90]	0.54	[0.33,0.88]
		p = 0.030		p = 0.005		p = 0.016		p = 0.013
**Anaemia**								
Hb (g/dl)^4^	1.11	[1.05,1.16]			1.11	[1.02,1.22]	1.14	[1.04,1.26]
		p<0.001				p = 0.022		p = 0.007

GEE-model based estimates with semi-robust confidence intervals.

IRR: Incidence rate ratio. CI_95_: 95% confidence intervals

1 Comparison level: Iliata 1. ^2^ Comparison level: January. ^3^ Average bed net usage as continuous variable. ^4^Per g/dl increase.

**Table 4:** Multivariate predictors of incidence of infections in 8 × 8/9 weeks intervals: *P. vivax* and *P. malariae* episodes of different parasite density.

	Pv all		Pv >500		Pv >2,000		Pm all	
	IRR	CI_95_	IRR	CI_95_	IRR	CI_95_	IRR	CI_95_
**Village** 1								
Ilaita 2	1.31	[0.75,2.31]	1.78	[0.88,3.62]	1.48	[0.60,3.65]		
Ilaita 3	1.97	[1.18,3.30]	2.75	[1.51,5.03]	2.52	[1.19,5.31]		
Ilaita 4	1.34	[0.87,2.06]	1.65	[0.91,3.01]	1.63	[0.77,3.43]		
Ilaita 5	0.62	[0.38,1.00]	0.47	[0.23,0.95]	0.32	[0.13,0.80]		
Ilaita 6	1.47	[0.80,2.58]	1.98	[1.00,3.94]	2.08	[0.89,4.88]		
Ilaita 7	1.31	[0.87,1.98]	1.46	[0.82,2.64]	1.10	[0.51,2.34]		
Ingamblis	0.56	[0.41,1.11]	0.54	[0.25,1.15]	0.40	[0.15,1.10]		
Kamanokor	0.85	[0.53,1.37]	0.90	[0.49,1.64]	0.91	[0.43,1.94]		
Sunuhu 1	0.78	[0.51,1.19]	0.96	[0.56,1.66]	0.80	[0.38,1.67]		
Sunuhu 2	0.71	[0.45,1.11]	0.63	[0.34,1.18]	0.69	[0.31,1.55]		
		p<0.001		p<0.001		p<0.001		
**Month^2^**								
March	0.51	[0.36,0.71]	0.60	[0.42,0.84]	0.49	[0.32,0.75]		
May	0.41	[0.28,0.58]	0.46	[0.32,0.67]	0.42	[0.26,0.67]		
July	0.39	[0.25,0.62]	0.62	[0.42,0.90]	0.45	[0.28,0.73]		
September	0.77	[0.50,1.21]	1.11	[0.78,1.58]	1.13	[0.73,1.76]		
November	0.69	[0.43,1.11]	0.90	[0.61,1.33]	0.76	[0.47,1.24]		
		p<0.001		p<0.001		p<0.001		
**Year (’07)**	0.66	[0.46,0.93]						
		p = 0.020						
**Age**								
linear	0.75	[0.64,0.87]	0.50	[0.42,0.59]	0.45	[0.36,0.57]	1.53	[1.13,2.06]
		p<0.001		p<0.001		p<0.001		p = 0.005
**Infection status** (at start of interval)								
Pv	1.44	[1.18,1.75]	1.44	[1.13,1.83]				
Pm		p<0.001		p<0.001			5.28	[2.91,9.58]
								p<0.001
**Prior Drug**	1.21	[1.01,1.45]	1.37	[1.09,1.71]	1.67	[1.25,2.24]	0.47	[0.23,0.94]
		p = 0.037		p = 0.007		p<0.001		p = 0.033
**ITN use**							0.51	[0.27,0.97]
								p = 0.041
**Anaemia**								
Hb (g/dl)			1.15	[1.03,1.28]	1.24	[1.08,1.43]		
				p = 0.013		p = 0.002		

GEE-model based estimates with semi-robust confidence intervals.

IRR: Incidence rate ratio. CI_95_: 95% confidence intervals

1 Comparison level: Iliata 1. ^2^ Comparison level: January. ^3^ Average bed net usage as continuous variable. ^4^Per g/dl increase.

There was highly significant spatial variation in the incidence of *P. falciparum* (Table 3, any density: Χ^2^ = 32.5, df  = 10, p<0.001 Pf >2500: Χ^2^ = 35.2, df  = 5, p<0.001) and *P. vivax* (Table 4, any density: Χ^2^ = 47.5, df  = 10, p<0.001, Pv >500: Χ^2^ = 56.5, df  = 10, p<0.001) but not *P. malariae* episodes (any density: Χ^2^ = 7.4.0, df  = 10, p = 0.69). Areas with high *P. falciparum* and high *P. vivax* incidence were significantly different with *P. falciparum* incidence rates highest in Ilaita 1, Ilaita 5, Sunuhu 1&2 and Kamankor, while *P. vivax* episodes were most frequent in villages near Ilaita health centre such as Ilaita 2–4 and Ilaita 6 & 7.

### Age Trends in Burden of *Plasmodium* Infections and Disease

**Figure 4:**
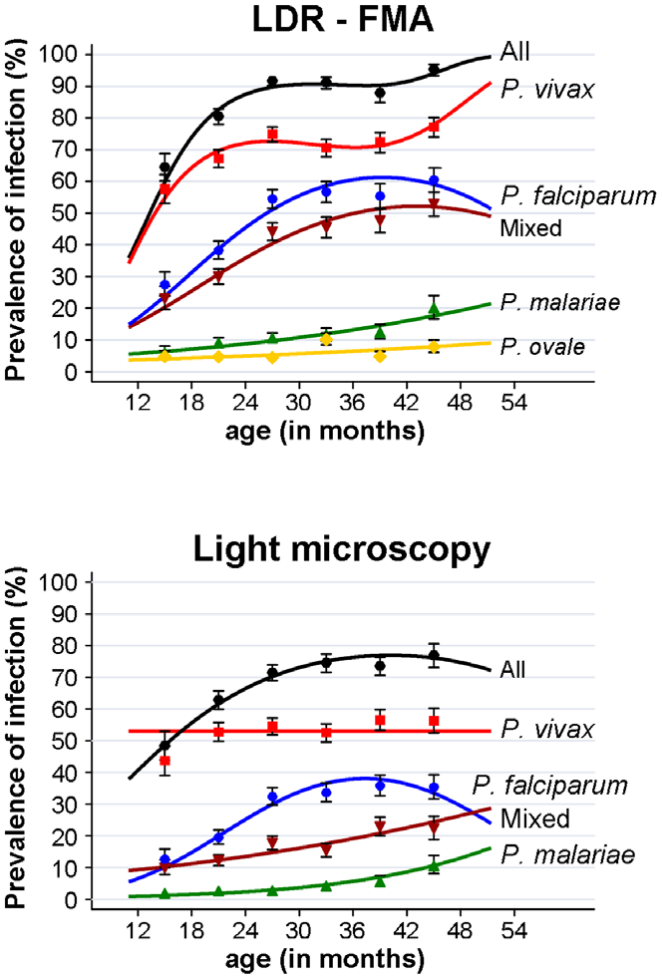
Age dependence of risk of malarial infections: age-prevalence at 7 cross-sectional survey time points with 2 consecutive bleeds 24 hrs apart. GEE model based estimates and semi-robust standard errors for age categories. Fitted lines: best fitting 1^st^, 2^nd^ or 3^rd^ degree polynomials of age as continuous variable.

The prevalence of malarial infections was significantly associated with the age of the child (Figure 4). Using only data from double bleed time points (i.e. weeks 9–60), the prevalence of *P. falciparum* was found to increase significantly (Χ^2^ = 35.3, df  = 2, p<0.001) from children <18 mth (LDR-FMA: 27.3%, LM: 12.3%) to children 18–23 mth (LDR-FMA 38.1%, LM: 19.3%) and children 24–29 mth (LDR-FMA 54.3%, LM 32.2%), with no further significant increase in older children (p = 0.24). For *P. vivax*, a similar age pattern was observed for LDR-FMA (<18 mth: 57.6%, 18–23 mth: 67.3%, ≥24 mth 73.5%, Χ^2^ = 12.5, df  = 2, p = 0.002) but not for LM diagnosed infections (Χ^2^ = 6.05, df  = 5, p = 0.30), while the prevalence of *P. malariae* continued to increase significantly across the entire age range (LDR-FMA: Χ^2^ = 15.3, df  = 5, p = 0.009, LM: Χ^2^ = 18.1, df  = 5, p = 0.003). Given the non-linear nature of these age trends, 1^st^, 2^nd^ and/or 3^rd^ order polynomials were used to model age trends in multivariate analyses (Figure 4, Table 1 & 2). Among children with LM positive infections, there was a significant decrease in geometric mean densities of *P. vivax* (<18 mth: 1624/µl, 18–23 mth: 989/µl, 24–29 mth: 614/µl, 30–35 mth: 484/µl, 36–41 mth: 475/µl, ≥42 mths 315/µl, Χ^2^ = 44.75, df  = 5, p<0.001) but not of *P. falciparum* infections (<18 mth: 8042/µl, 18–23 mth: 2350/µl, 24–29 mth: 4744/µl, 30–35 mth: 3576/µl, 36–41 mth: 3228/µl, ≥42 mths 1353/µl, Χ^2^ = 10.80, df  = 5, p = 0.055). Comparable patterns were observed when only data from the 1^st^ bleed were considered.

Among infected children, there was a significantly higher prevalence of febrile symptoms with *P. falciparum* (39.1%, OR  = 3.49, CICI_95_[2.48, 7.40], p<0.001) and mixed *P. falciparum* / *P. vivax* infections (22.6%, OR  = 1.58, CI_95_[1.18, 2.12], p = 0.002) compared to *P. vivax* only (15.5%) infections. Prevalence of febrile symptoms decreased significantly with age in children infected with *P. vivax* (OR (per year increase in age)  = 0.57, CI_95_[0.54, 0.85], p = 0.001) but not in *P. falciparum* or mixed infections (OR  = 0.97, CI_95_[0.77, 1.22], p = 0.77).

**Figure 5:**
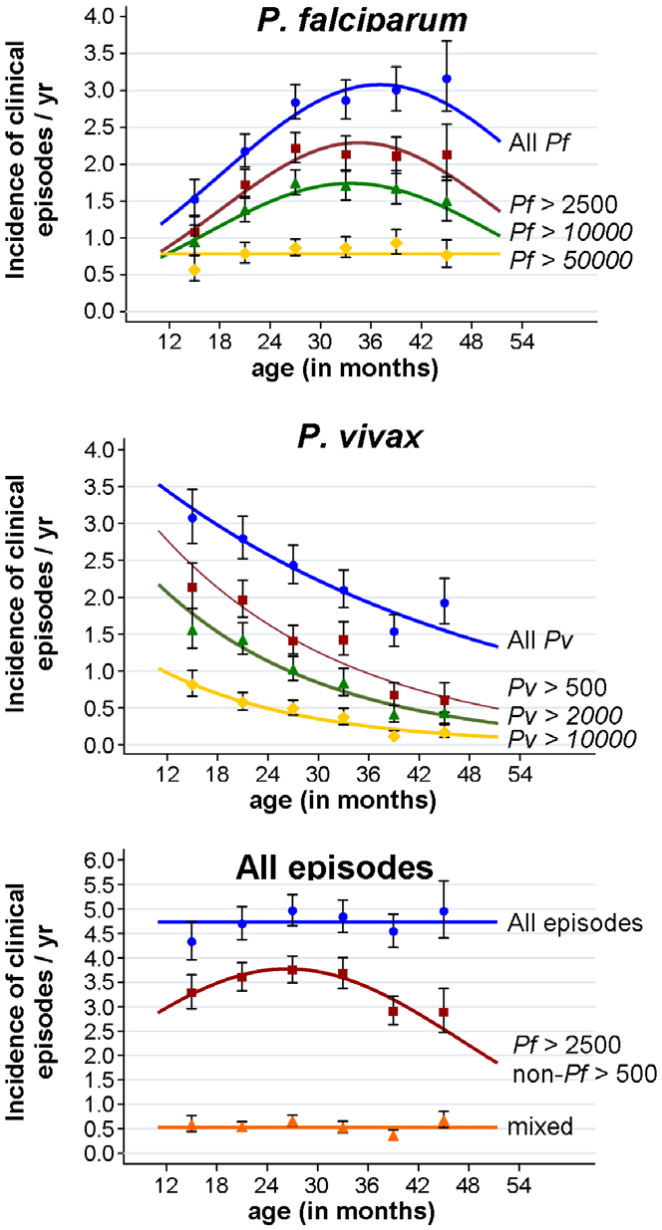
Age dependence of risk of malarial illness: Effect of age at start of 8/9 weeks follow-up period on incidence of malarial illness with different parasite cut-offs. GEE model based estimates and semi-robust standard errors for age categories. Fitted lines: best fitting 1^st^, 2^nd^ or 3^rd^ degree polynomials of age as continuous variable.

Although the overall incidence of malaria episodes did not vary greatly with age (Χ^2^ = 9.4, df  = 5, p = 0.095), the incidence of *P. falciparum* and *P. vivax* episodes were strongly age dependent, albeit with opposing trends (Figure 5). In parallel with prevalence of infection, incidence of *P. falciparum* episodes rose significantly (any density: Χ^2^ = 13.2, df  = 2, p = 0.001, Pf >2500: Χ^2^ = 11.2, df  = 2, p = 0.004) from children <18 mth (any density: IR 1.50, Pf >2500: IR 1.06) to children 18–23 mth (any density: IR 2.15, Pf >2500: IR 1.71) and children 24–29 mth (any density: IR 2.81, Pf >2500 IR 2.20), with no further significant increase in incidence thereafter (any density: Χ^2^ = 0.58, df  = 3, p = 0.90, Pf >2500: Χ^2^ = 0.23, df  = 3, p = 0.97). No significant difference between age groups was observed for high and very high density episodes (Pf >10,000: Χ^2^ = 7.5, df  = 5, p = 0.19, Pf >50,000: Χ^2^ = 1.52, df  = 5, p = 0.47). The incidence of *P. vivax* episodes, however, decreased significantly with age throughout the entire age range (any density: Χ^2^ = 19.0, df  = 5, p = 0.002, Pv >500: Χ^2^ = 29.1, df  = 5, p<0.001) from 3.07 (any density) and 2.13 (Pv >500) episodes / child / per year in children <18 mths to 1.52 (any density) in children 36–41 mths and 0.59 (Pv >500) in children ≥42 mths. The observed decreases with age were log-linear with decreasing incidence rate ratios (IRR, per year increase in age) with increasing levels of parasitaemia (any density, crude IRR  = 0.75, CI_95_[0.65,0.85], p<0.001; Pv >500, IRR  = 0.59, CI_95_[0.49,0.71], p<0.001; Pv >2000, IRR = 0.55, CI_95_[0.44,0.69], Pv >10000, IRR = 0.50 CI_95_[0.38,0.71], p<0.001). The incidence of *P. malariae* episodes increased significantly with age (any density: IRR  = 1.66, CI_95_ [1.22, 2.25], p<0.001). These age effects remained significant when adjusted for other factors (see Table 3&4).

### Effects of Insecticide Treated Nets

**Figure 6:**
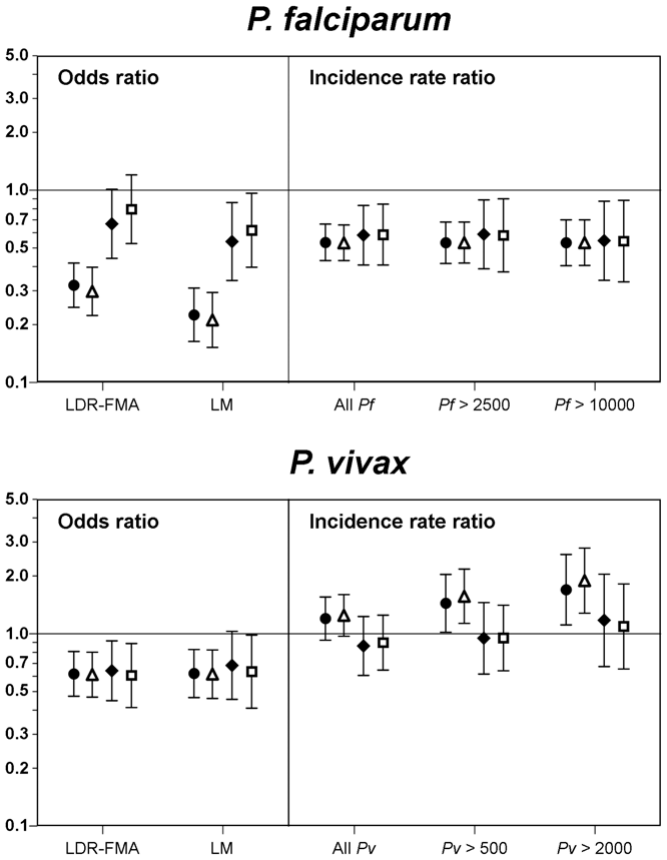
Association of personal bed net use on risk of malarial infections and disease. Closes circles: univariate estimates, open triangle: adjusted for age and time trends, closed diamonds: age, time and village adjusted, open squares: estimates from best fitting model (see Table 1 & 2). All estimates from GEE model with semi-robust standard errors.

Children who slept under insecticide treated bed nets (ITNs) had significantly fewer malarial infections (Tables 1 and 2). However, there were significant differences in ITN use (F_10,252_  = 22.90, p<0.001) among villages with >80% usage in Ilaita 4 & 5, 58–80% usage in the other Ilaita villages while in the more remote villages only 0–35% of children reported sleeping under a bed net. This unequal spatial distribution complicates the assessment of the association of ITN use with risk of malarial infections and disease. Adjusted for age and time trends, ITN usage was associated with a significant reduction in prevalence of malarial infections with any of the 4 malarial species diagnosed by LDR (Figure 6, Adjusted Odd Ratio (AOR): Pf: 0.30, p<0.001; Pv: 0.61, p<0.001; Pm: 0.62, p = 0.027; Po: 0.52, p = 0.015) and LM (AOR: Pf: 0.21, p<0.001; Pv: 0.61, p = 0.001; Pm: 0.29, p = 0.002). Further adjusting for difference in prevalence among villages significantly reduced the association of ITN usage with prevalence of *P. falciparum* (AOR: LDR: 0.67, p = 0.054; LM: 0.54, p = 0.009) and *P. malariae* (AOR: LDR: 1.18, p = 0.508; LM: 0.65, p = 0.345) but not of *P. vivax* (AOR: LDR: 0.64, p = 0.015; LM: 0.68, p = 0.066) and *P. ovale* (AOR: LDR: 0.49, p = 0.070). Adjustment for other factors did not significantly alter the associations of ITN with prevalence of malarial infection (Figure 6, Tables 1 & 2).

Frequent use of ITNs also provided significant protection against *P. falciparum* and *P. malariae* but not against *P. vivax* episodes (Tables 3 & 4). Adjusted for age and time trends, ITN usage was associated with a significant reduction in incidence of *P. falciparum* (Figure 6, any density, Pf >2500 & Pf >10000: all aIRRs  = 0.53, p<0.001) and *P. malariae* episodes (any density: aIRR  = 0.47, p<0.001) but a significant increase in the risk of moderate to high density *P. vivax* episodes (any density: aIRR  = 1.24, p = 0.09; Pv >500: aIRR  = 1.56, p = 0.007; Pv >2000: aIRR  = 1.88, p = 0.001). When also adjusted for villages of residence, the association of ITN usage with increased risk of *P. vivax* episodes was no longer seen (any density: aIRR  = 0.86, p = 0.41; Pv >500: aIRR  = 0.94, p = 0.80; Pv >2000: aIRR  = 1.17, p = 0.58), whereas the effect of ITNs on risk of *P. falciparum* (any density: aIRR  = 0.58, p = 0.003; Pf >2500: aIRR  = 0.59, p = 0.011; Pf >10000: aIRR  = 0.54, p = 0.012) and *P. malariae* (any density: aIRR  = 0.64, p = 0.28) was unchanged. Adjustment for other factors did not significantly alter the associations of ITN with risk of malaria episodes (Figure 6, Tables 3 &4).

### Other Predictors for *Plasmodium* Infection and Disease

Additional independent predictors for the presence of *Plasmodium* infections at cross-sectional bleeds included confirmed antimalarial treatment less than 4 weeks prior, reported febrile illness in preceding 2 weeks, presence of a febrile illness and/or enlarged spleen and hemoglobin (Hb) levels (Table 1 & 2). Recent antimalarial treatments were associated with a significant decrease in risk of *P. vivax* by both LDR and LM but an increase in risk of LDR but not LM positive *P. falciparum*, while reported febrile illness in the previous 2 weeks was associated with a significant decrease in risk of LDR positive *P. vivax* and LDR and LM positive *P. malariae* infections. The presence of febrile symptoms was positively associated with *P. falciparum* infections while enlarged spleens were found more often in children infected with *P. falciparum* or *P. malariae*. Higher Hb levels were associated with a decreased prevalence of *P. falciparum* and *P. malariae* (LM only) but an increased prevalence of *P. vivax* infections.

Multivariate predictors of risk of clinical malaria also included infection status, recent antimalarial treatment and Hb levels at the start of the 8 week interval (Tables 3 & 4). For all types of clinical episodes except high density *P. vivax* (Pv >2,000), the presence of an infection at the start of the interval was associated with a significant increase in risk of clinical episodes with the same *Plasmodium* species during the following 8–9 weeks (adjusted incidence rate ratio (aIRR): Pf 1.23–1.43, p = 0.028–0.002; Pv 1.44, p<0.001; Pm 5.28 p<0.001). Treatment with an antimalarial no more the 4 weeks prior to the start of the follow-up period resulted in a decrease in the incidence of clinical disease for low to moderate density episodes of *P. falciparum* and *P. malariae*, but not for *P. vivax* and high density *P. falciparum* episodes (Table 3 & 4). Increasing Hb levels on the other hand were associated with an increase in the risk of *P. falciparum* and *P. vivax* episodes (aIRR (per g increase in Hb): Pf>2500: 1.11, p = 0.022; Pf >10,000: 1.14 p = 0.007; Pv >500: 1.15, p = 0.013, Pv >2000: 1.24, p = 0.002).

## Discussion

The present study shows a significantly faster acquisition of clinical immunity to *P. vivax* compared to *P. falciparum* in Papua New Guinean children aged 1–4 yrs of age. Despite comparable numbers of clinical episodes with *P. falciparum* and *P. vivax* in this age group, there was a highly significant drop in the incidence of uncomplicated *P. vivax* episodes with increasing age but little signs of any acquisition of protective immunity against uncomplicated *P. falciparum* illness. On the contrary, the incidence of *P. falciparum* illness increased in the 2^nd^ year and remained constant during the 3^rd^ and 4^th^ years of life.

While the rise in the incidence of *P. falciparum* episodes went hand in hand with a rise in the prevalence of *P. falciparum* infection, a moderate increase in the prevalence of *P. vivax* infections was observed despite a 72% drop in the incidence of *P. vivax* episodes with a density exceeding a pyrogenic threshold of 500 parasites/µl in children younger than 18 mth compared to those older than 42 mth of age. Both the geometric mean density of *P. vivax* infections and the risk of febrile symptoms associated with the presence of *P. vivax* infections decreased significantly with age, indicating an increasing ability of older children to control *P. vivax* density at levels below the pyrogenic threshold. Both the density of *P. falciparum* infection and prevalence of febrile symptoms associated with such infections did not vary significantly within the age range covered by this study, indicating that even at 3–4 yrs of age the ability to effectively control *P. falciparum* parasitaemias has not yet been acquired.

The current study thus confirms and extends the finding of a significantly faster rate of immune acquisition to *P. vivax*, as described by Michon et al [5], to a different PNG population; from children 5–14 yrs with a high degree of clinical immunity to *P. vivax* to much younger children that are still at high risk of both clinical *P. falciparum* and *P. vivax* disease. The patterns found in the present cohort are also consistent with those observed in a cohort of children under 10 years conducted in neighbouring Vanuatu [12]. Despite a lower transmission than *P. falciparum*, Maitland et al. also observed that the incidence of clinical *P. vivax* episodes peaked in younger children (Pv: 1–2 yrs IR  = 1.15 episodes per child per year vs Pf: 3–4 yrs IR  = 0.93) and that parasite densities in both symptomatic and asymptomatic *P. vivax* but not *P. falciparum* infections decreased with age. Even at very low transmission among Karen communities along the Thai-Burmese border, *P. vivax* prevalence and incidence peak in significantly younger age groups than *P. falciparum* [33]. It is thus likely that the faster acquisition of immunity to *P. vivax* (compared to *P. falciparum*) is a general phenomenon in people with life-long natural exposure across a wide range of malaria transmission intensities. Further studies in Thailand and Sri Lanka corroborate this view [34], [35].

The reasons for the faster acquisition of clinical immunity to *P. vivax* are not well understood. It may simply be a function of the higher burden of infection and illness in very early life. Two specific aspects of *P. vivax* biology are likely to have contributed to the higher overall prevalence of *P. vivax* infection and the lack of a clear seasonality in the present cohort: its ability to re-establish blood stage infections through relapses from long-lasting liver-stages and its high transmissibility. *P. vivax* is known to produce gametocytes as soon as a blood stage infection is established and at low parasite density [36]. In PNG, the prevalence of *P. vivax* gametocytes can exceed that of *P. falciparum* even when the prevalence of asexual stages is lower[37]. Furthermore, *P. vivax* is more infective to mosquitoes fed on blood collected at village population surveys and comparable when fed on blood from gametocyte positive outpatients [37]. In addition, younger, early biting mosquitoes tend be more commonly infected by *P. vivax* than *P. falciparum* [38] in particular in the presence of ITNs [39]. Such an increased transmissibility would facilitate both the early establishment of *P. vivax* infection in young children and allow sustained transmission through periods of reduced transmission potential. In PNG, *P. vivax* is usually the most common infection in infants and toddlers [9], [10]. Once infections are established, relapses from long-lasting liver-stages will occur regularly and contribute significantly to a high rate of blood stage infections. In a recent drug trial between 27% and 87% of PNG children 6–60 months of age treated for *P. vivax* or *P. falciparum* episodes had recurrent asexual *P. vivax* parasitaemia within 42 days [40].

Data from US malaria therapy patients who received two *P. vivax* infections indicate that immunity against re-infection with the homologous *P. vivax* strain is acquired very rapidly. Both the frequency and intensity of febrile episodes, as well as peak and mean parasite densities, decrease by more than 80% during the first 20 days of parasitaemia.[16]. The frequency of febrile episodes also decreased by 48–55% in re-infections with a heterologous strain with equal peak parasitaemias but lower mean parasitaemias indicating the presence of a limited level of cross-immunity. In patients infected with *P. falciparum*, immunity to homologous *P. falciparum* re-infections developed more slowly while no reduction in the development of fever or higher density parasitaemia was seen upon heterologous challenge [17]. Similar differences in the development of strain-specific and strain transcending immunity were also observed in Romanian patients [15]. The lower risk of febrile symptoms associated with *P. vivax* infection during the dry season, when many infections are likely to be due to relapses rather than primary infections, and consequently the different seasonal trends of *P. vivax* infections and disease observed in the present cohort, indicate that similar differences in immune acquisition may also occur under conditions of natural exposure.

The incidence of clinical episodes with any malarial species during an 8 or 9 week interval was positively associated with the presence of parasitaemia (with same species) at the start of the interval, indicating that either ‘asymptomatic’ infections detected during cross-sectional surveys may go on to become symptomatic later on and/or that there is significant heterogeneity in transmission among participants (i.e. children with an infection during the cross-sectional survey are more likely to acquire new infections with the same species than uninfected children). Detailed genotyping of *P. falciparum* and *P. vivax* infections will be needed to determine the respective contribution of persistent and new infections to the burden of disease. The incidence of *P. falciparum* and *P. malariae* episodes was negatively associated with receiving an antimalarial drug less than 4 weeks prior, but the incidence of *P. vivax* episode was higher in children with a recent treatment. This difference is most likely due to the very high rate of recurrent *P. vivax* parastitaemia 4–6 weeks following treatment with Coartem® or Amodiaquine plus sulphadoxine-pyrimethamine [40].

Children who reported always sleeping under insecticide treated bed nets (ITN) experienced 42% and 53% fewer episodes (adjusted for other factors) of *P. falciparum* and *P. malariae*, respectively than children who never reported using ITNs. Use of ITNs was also associated with a significant decrease in the prevalence of light microscopy positive *P. falciparum* but not with LDR-FMA positive *P. falciparum* or *P. malariae* infections. These results are remarkably similar to those obtained in randomised control trials in Africa where sleeping under an ITN was associated with a 50% decrease in risk of uncomplicated *P. falciparum* illness and a 16% decrease in prevalence of *P. falciparum* infection [41].

As in a randomised control trial in Thailand [42], the incidence of clinical *P. vivax* malaria episodes was not significantly affected by bed net use. The prevalence of *P. vivax* infections was however significantly lower in children using ITNs. This paradoxical pattern may be related to the unequal spatial distribution of ITNs in the study area. ITNs are most common in villages that are close to Ilaita health centre and thus have better access to health care. These villages also have a higher incidence of *P. vivax* illness but lower prevalence of *P. vivax* infections (by LDR-FMA) than villages further away from the health centre.

The ease of access to health care may have a differential impact on health seeking behaviour in relation to *P. falciparum* and *P. vivax* illness. With increasing immunity the duration and severity of fevers associated with *P. vivax* infection tend to decrease rapidly, in particular for recurrences with the same strain either through re-infection [16] or relapse. If access to health care is nearby, parents seek treatment more quickly and for less severe symptoms than if they have to walk several hours [43], [44]. Parents of children in more remote villages may thus have delayed treatment seeking for low level fevers associated with *P. vivax* infection (e.g. from relapsing parasites) until the study team next visited the villages. By that time many of the symptoms would have resolved through control of the infection by the immune system, negating the need for antimalarial treatment. Due to the lower immunity to *P. falciparum*, few *P. falciparum* episodes would have been mild and self-limiting, prompting parents to more readily seek treatment even in more remote villages and/or for symptoms to persist until the next study team visit. These differences in treatment seeking are well in line with the fact that *P. vivax* episodes were more commonly detected amongst cases presenting to the health centre, while *P. falciparum* episodes dominated during study visits (Lin & Mueller, personal communication). A potential beneficial effect of ITNs on incidence of *P. vivax* episodes would therefore have been off-set by more active treatment seeking and easy access to treatment in villages with high bed net usage. Conversely, frequent treatment of *P. vivax* episodes is likely to have limited the establishment of chronic, asymptomatic infections, thus resulting in a bias towards a lower prevalence of *P. vivax* infections in villages near the health centre.

The present study thus confirms the earlier findings of significant differences in the acquisition of clinical immunity to *P. falciparum* and *P. vivax* in children 5–14 yrs of age [5] in a cohort of younger Papua New Guinean children (1–4 years) who experience high levels of clinical illness with either *Plasmodium* species. Despite similar overall incidences of clinical disease, the incidence of *P. vivax* episodes starts to decrease significantly in the 2^nd^ year of life when the incidence of *P. falciparum* episodes is still rising. The acquisition of clinical immunity to *P. vivax* is characterized by an increasing ability to control *P. vivax* parasitaemia that is not apparent in *P. falciparum* infections. In the age group studied (i.e. 1–4 yrs), there is thus clear evidence for the acquisition of clinical immunity to *P. vivax* but not yet to *P. falciparum*.

The exact nature of the processes underlying the observed difference in immune acquisition remain to be elucidated but differences in the interaction of parasite with the human red cell are likely to play an important role. *P. falciparum* can avoid clearance by the spleen through sequestration of infected RBC into microvasculature via binding of variant surface antigens (VSA) to a variety of host receptors [45], [46]. The continued acquisition of immune responses against a broad range of VSAs is thought to be critical for acquiring protective immunity against *P. falciparum* illness and infection [22], [47]. *P. vivax* does not have a gene family homologous to *Pf*EMP1, the most important group of VSAs in *P. falciparum* [48]. The *P. vivax* multi-gene *vir* family is not expressed clonally and different *vir* proteins are found concurrently both on the RBC surface and in other parasite organs [49]. Although it has been hypothesised that vir-gene are involved in avoidance of splenic clearance, the exact function of *vir*-proteins remains to be determined[49].

*P. falciparum* is also known to use a variety of host receptors for invasion of host red blood cells [50]. Despite a recent report of *P. vivax* invading Duffy negative RBC (Ménard, Barnadas & Zimmerman, personal communication), efficient invasion of *P. vivax* seems to require binding of the *Pv*DBP to the Duffy antigen. Antibodies blocking this interaction have been shown to confer a high level of protection against *P. vivax* infection [26]. However, high levels of *Pv*DBP blocking antibodies are not commonly found even among adults with complete clinical immunity to *P. vivax*, indicating that immune responses directed against other parasites antigens (or against other parts of the *Pv*DBP protein) may be more important for the acquisition of immunity to *P. vivax* under natural exposure. If and how any differences in antigenic diversity in such key antigens contribute to differential immune acquisition between *P. falciparum* and *P. vivax* remains to be determined.

Detailed, comparative studies aimed at understanding these differences in parasite biology and host immune responses to *P. falciparum* and *P. vivax* are on-going. Such comparative studies on immune acquisition to both *P. falciparum* and *P. vivax* species that combine a rigorous, longitudinal study design with in-depth immunological and parasitological assessments will provide valuable insights into differences in immune targets and mechanisms underlying acquisition of clinical immunity that may inform malaria vaccine development and testing.
